# Supplementary material for: Nitrogen Limitation of Intense and Toxic Cyanobacteria Blooms in Lakes within Two of the Most Visited Parks in the USA: The Lake in Central Park and Prospect Park Lake
Source: Toxins (Basel). 2022 Oct 6;14(10):684. doi: 10.3390/toxins14100684 (PMC9612084; doi:10.3390/toxins14100684)
Supplement: Supplementary file 1 [file toxins-14-00684-s001.zip › toxins-1925304-supplementary.pdf]

toxins

Nitrogen Limitation of Intense and Toxic Cyanobacteria Blooms in Lakes within Two of the Most Visited Parks in the USA: The Lake in Central Park and Prospect Park Lake

Jacob M. Flanzenbaum, Jennifer G. Jankowiak, Jennifer A. Goleski, Rebecca M. Gorney and Christopher J. Gobler

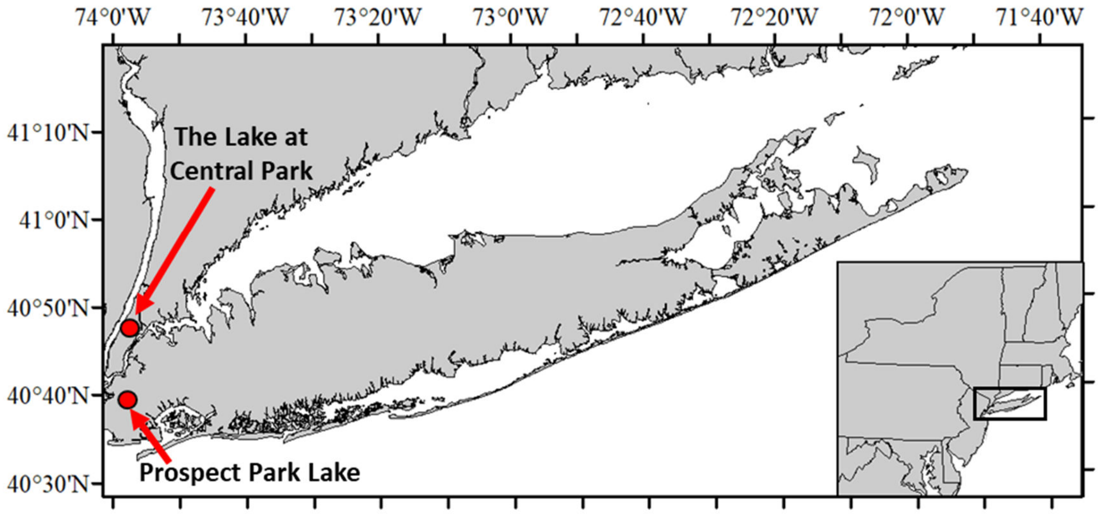

Figure S1. Map of the two study sites: The Lake in Central Park and Prospect Park Lake in New York City, NY, USA.

Table S1. Genera of microscopically identified most abundant cyanobacteria in LCP, 2015-2019.

Date	Cyanobacteria taxa	Date	Cyanobacteria taxa
**6/3/2015**	*Microcystis*	**7/10/2017**	*Microcystis, Planktothrix*
**6/10/2015**	*Microcystis*	**7/17/2017**	*Microcystis, Planktothrix*
**6/16/2015**	*Microcystis*	**7/24/2017**	*Microcystis*
**6/24/2015**	*Microcystis*	**7/31/2017**	*Microcystis*
**6/30/2015**	*Microcystis*	**8/7/2017**	*Microcystis, Dolichospermum*
**7/7/2015**	*Microcystis*	**8/14/2017**	*Microcystis*
**7/14/2015**	*Microcystis*	**8/21/2017**	*Microcystis*
**7/21/2015**	*Microcystis*	**8/28/2017**	*Microcystis*
**7/28/2015**	*Microcystis*	**9/5/2017**	*Microcystis*
**8/3/2015**	*Microcystis*	**9/18/2017**	*Microcystis*
**8/26/2015**	*Microcystis*	**9/26/2017**	*Microcystis, Dolichospermum, Aphanizomenon*
**8/26/2015**	*Microcystis*	**9/26/2017**	*Microcystis, Dolichospermum, Aphanizomenon*
**9/3/2015**	*Microcystis*	**10/2/2017**	*Microcystis, Dolichospermum, Aphanizomenon*
**9/3/2015**	*Microcystis*	**10/10/2017**	*Microcystis*
**9/10/2015**	*Microcystis*	**10/16/2017**	*Microcystis, Aphanizomenon*
**9/10/2015**	*Microcystis*	**10/23/2017**	*Microcystis, Aphanizomenon*
**9/24/2015**	*Microcystis*	**11/7/2017**	*Microcystis, Aphanizomenon*
**9/24/2015**	*Microcystis*	**6/4/2018**	*Microcystis*
**9/30/2015**	*Microcystis*	**6/11/2018**	*Microcystis*
**9/30/2015**	*Microcystis*	**6/25/2018**	*Microcystis, Dolichospermum*
**10/21/2015**	*Microcystis*	**7/2/2018**	*Microcystis, Dolichospermum, Aphanizomenon*
**10/30/2015**	*Microcystis*	**7/9/2018**	*Microcystis, Dolichospermum, Planktothrix, Aphanizomenon*
**6/15/2016**	*Microcystis*	**7/16/2018**	*Microcystis, Planktothrix*
**6/22/2016**	*Microcystis*	**7/23/2018**	*Microcystis*
**6/30/2016**	*Microcystis*	**7/30/2018**	*Microcystis*
**7/6/2016**	*Microcystis*	**8/6/2018**	*Microcystis*
**7/13/2016**	*Microcystis*	**8/13/2018**	*Microcystis*
**7/20/2016**	*Microcystis*	**8/20/2018**	*Microcystis*
**7/27/2016**	*Microcystis*	**8/27/2018**	*Microcystis*
**8/2/2016**	*Microcystis*	**9/4/2018**	*Microcystis*
**8/10/2016**	*Microcystis, Oscillatoria*	**9/10/2018**	*Microcystis, Planktothrix*
**8/17/2016**	*Microcystis*	**9/17/2018**	*Microcystis, Planktothrix*
**8/24/2016**	*Microcystis*	**9/24/2018**	*Microcystis*
**8/29/2016**	*Microcystis*	**10/1/2018**	*Microcystis*
**9/8/2016**	*Microcystis*	**10/9/2018**	*Microcystis, Dolichospermum*
**9/12/2016**	*Microcystis*	**5/20/2019**	*Microcystis, Aphanizomenon*
**9/21/2016**	*Microcystis*	**6/3/2019**	*Microcystis, Aphanizomenon*
**9/28/2016**	*Microcystis*	**6/10/2019**	*Microcystis*
**10/5/2016**	*Microcystis*	**6/17/2019**	*Microcystis, Aphanizomenon*
**10/12/2016**	*Microcystis*	**6/24/2019**	*Microcystis*
**10/19/2016**	*Microcystis, Dolichospermum*	**7/8/2019**	*Microcystis*
**10/26/2016**	*Microcystis, Planktothrix*	**7/22/2019**	*Microcystis, Planktothrix, Dolichospermum*
**6/21/2017**	*Microcystis, Planktothrix*	**8/5/2019**	*Microcystis, Planktothrix*
**6/26/2017**	*Microcystis, Planktothrix*	**8/12/2019**	*Microcystis, Planktothrix*
**7/5/2017**	*Microcystis*		

Table S2. Genera of microscopically identified most abundant cyanobacteria in PPL, 2015-2019.

Date	Cyanobacteria taxa	Date	Cyanobacteria taxa
**[illegible]015**	*Microcystis, Dolichospermum, Aphanizomenon*	**7/31/2017**	*Microcystis, Planktothrix*
**[illegible]2015**	*Microcystis, Dolichospermum, Aphanizomenon*	**8/7/2017**	*Microcystis, Planktothrix*
**[illegible]2015**	*Microcystis, Dolichospermum, Aphanizomenon*	**8/14/2017**	*Microcystis, Planktothrix*
**[illegible]2015**	*Microcystis, Dolichospermum, Aphanizomenon*	**8/21/2017**	*Microcystis, Planktothrix*
**[illegible]015**	*Microcystis, Dolichospermum*	**8/28/2017**	*Microcystis, Planktothrix*
**[illegible]015**	*Microcystis, Dolichospermum*	**9/5/2017**	*Microcystis, Planktothrix*
**[illegible]2015**	*Microcystis*	**9/11/2017**	*Microcystis, Planktothrix*
**[illegible]2015**	*Microcystis, Dolichospermum*	**9/18/2017**	*Microcystis, Planktothrix*
**[illegible]2015**	*Microcystis, Dolichospermum*	**9/25/2017**	*Microcystis, Dolichospermum, Aphanizomenon*
**[illegible]015**	*Microcystis, Dolichospermum*	**9/25/2017**	*Microcystis, Dolichospermum, Aphanizomenon*
**[illegible]2015**	*Microcystis*	**10/2/2017**	*Microcystis, Dolichospermum, Planktothrix*
**[illegible]2015**	*Microcystis*	**10/10/2017**	*Microcystis, Dolichospermum, Planktothrix*
**[illegible]015**	*Microcystis*	**10/16/2017**	*Microcystis, Planktothrix*
**[illegible]2015**	*Microcystis*	**10/23/2017**	*Microcystis, Planktothrix*
**[illegible]015**	*Microcystis, Dolichospermum, Aphanizomenon*	**5/21/2018**	*Microcystis, Dolichospermum*
**[illegible]2015**	*Microcystis, Planktothrix*	**5/29/2018**	*Microcystis, Dolichospermum*
**[illegible]2015**	*Microcystis, Planktothrix*	**6/4/2018**	*Microcystis, Dolichospermum*
**[illegible]2015**	*Planktothrix*	**6/11/2018**	*Microcystis, Dolichospermum, Aphanizomenon*
**6/14/2016**	*Microcystis*	**6/18/2018**	*Microcystis*
**6/21/2016**	*Microcystis, Planktothrix*	**6/25/2018**	*Microcystis, Dolichospermum*
**6/28/2016**	*Microcystis*	**7/9/2018**	*Microcystis*
**7/6/2016**	*Microcystis, Planktothrix*	**7/16/2018**	*Microcystis, Dolichospermum, Planktothrix*
**7/11/2016**	*Microcystis, Planktothrix*	**7/23/2018**	*Microcystis, Dolichospermum, Planktothrix*
**7/20/2016**	*Microcystis, Dolichospermum, Planktothrix*	**7/30/2018**	*Microcystis, Planktothrix*
**7/28/2016**	*Microcystis*	**8/6/2018**	*Microcystis, Planktothrix*
**8/2/2016**	*Microcystis, Planktothrix*	**8/13/2018**	*Microcystis, Dolichospermum, Aphanizomenon*
**8/8/2016**	*Microcystis*	**8/20/2018**	*Microcystis, Planktothrix*
**8/19/2016**	*Microcystis, Planktothrix*	**8/27/2018**	*Microcystis, Planktothrix*
**8/23/2016**	*Microcystis, Planktothrix*	**9/4/2018**	*Aphanizomenon*
**8/29/2016**	*Microcystis, Planktothrix*	**9/10/2018**	*Planktothrix*
**9/6/2016**	*Microcystis, Planktothrix*	**9/17/2018**	*Microcystis, Planktothrix*
**9/12/2016**	*Microcystis, Planktothrix*	**9/24/2018**	*Planktothrix*
**9/19/2016**	*Microcystis, Dolichospermum, Aphanizomenon*	**10/1/2018**	*Microcystis, Planktothrix*
**9/27/2016**	*Microcystis, Planktothrix*	**10/9/2018**	*Microcystis, Planktothrix*
**10/3/2016**	*Microcystis, Planktothrix*	**10/15/2018**	*Microcystis, Planktothrix*
**10/11/2016**	*Planktothrix*	**10/29/2018**	*Microcystis, Planktothrix*
**10/17/2016**	*Microcystis, Planktothrix*	**5/20/2019**	*Aphanizomenon*
**10/24/2016**	*Microcystis, Planktothrix*	**5/28/2019**	*Microcystis*
**10/31/2016**	*Microcystis, Planktothrix*	**6/4/2019**	*Microcystis*
**11/7/2016**	*Dolichospermum, Planktothrix*	**6/10/2019**	*Dolichospermum*
**11/14/2016**	*Microcystis, Planktothrix*	**6/17/2019**	*Microcystis, Aphanizomenon, Dolichospermum*
**5/22/2017**	*Microcystis, Dolichospermum*	**6/25/2019**	*Microcystis, Aphanizomenon, Dolichospermum*
**5/30/2017**	*Microcystis, Dolichospermum*	**7/15/2019**	*Microcystis, Aphanizomenon*
**6/6/2017**	*Microcystis, Dolichospermum*	**7/22/2019**	*Microcystis, Planktothrix*
**6/12/2017**	*Microcystis, Dolichospermum*	**7/29/2019**	*Microcystis, Aphanizomenon*
**6/19/2017**	*Microcystis*	**8/5/2019**	*Planktothrix, Dolichospermum*
**6/26/2017**	*Microcystis, Dolichospermum, Aphanizomenon*	**8/12/2019**	*Microcystis, Planktothrix*
**7/5/2017**	*Microcystis, Dolichospermum, Woronichinia*	**8/19/2019**	*Microcystis, Planktothrix*
**7/12/2017**	*Microcystis, Planktothrix, Oscillatoria*	**8/26/2019**	*Microcystis, Planktothrix*
**7/12/2017**	*Microcystis, Dolichospermum, Aphanizomenon, Oscillatoria*	**9/23/2019**	*Microcystis, Planktothrix*
**7/17/2017**	*Microcystis, Aphanizomenon*	**10/7/2019**	*Microcystis, Dolichospermum, Planktothrix*
**7/24/2017**	*Microcystis, Dolichospermum, Planktothrix*		

Table S3. Genera of microscopically identified most abundant cyanobacteria in LCP and PPL, 2020.

Date	Cyanobacteria taxa (LCP)	Date	Cyanobacteria taxa (PPL)
**6/17/2020**	*Microcystis*	**6/24/2020**	*Microcystis, Aphanizomenon, Dolichospermum*
**7/1/2020**	*Microcystis, Dolichospermum*	**7/8/2020**	*Microcystis, Planktothrix*
**7/15/2020**	*Microcystis*	**7/22/2020**	*Microcystis*
**7/29/2020**	*Microcystis, Planktothrix, Aphanizomenon*	**7/29/2020**	*Microcystis*
**8/12/2020**	*Microcystis*	**8/5/2020**	*Microcystis, Planktothrix*
**8/26/2020**	*Microcystis*	**8/19/2020**	*Microcystis, Planktothrix, Dolichospermum*
**9/9/2020**	*Microcystis, Dolichospermum*	**9/3/2020**	*Microcystis, Planktothrix, Dolichospermum*
**9/21/2020**	*Microcystis*	**9/17/2020**	*Microcystis*
**10/7/2020**	*Microcystis, Planktothrix*	**10/1/2020**	*Microcystis*
**10/22/2020**	*Microcystis*	**10/15/2020**	*Microcystis, Dolichospermum, Aphanizomenon*
		**10/30/2020**	*Microcystis, Planktothrix*

Table S4. Spearman Rank Order Correlation of environmental parameters measured in LCP and PPL in 2020. Numbers appearing vertically in order are correlation coefficient, *p*-value, and number of samples. MC is microcystin, Cyano is cyanobacterial biomass measured fluorometrically, Green is green algal biomass measured fluorometrically, Chla is chlorophyll *a* measured fluorometrically, 16S is cyanobacteria measured via qPCR, mcyE is levels of the *mcyE* gene measured via qPCR, stxA is levels of the *stxA* gene measured via qPCR, T is temperature, DO is dissolved oxygen, NOx is nitrate/nitrite, NH4 is ammonium, TN is total N, PO4 is orthophosphate, TP is total P, DIN:DIP, and TN:TP are nutrient ratios. Units for all parameters are shown in the main figures of the manuscript.

	**MC**	**Cyano**	**Green**	**Chla**	**16S**	**mcyE**	**sxtA**
Date	0.128, 0.585, 20	0.0468, 0.837, 21	-0.0717, 0.754, 21	-0.142, 0.535, 21	0.0602, 0.797, 20	0.220, 0.347, 20	0.0980, 0.676, 20
MC		0.275, 0.236, 20	0.574, 0.00814, 20	0.559, 0.0104, 20	-0.423, 0.0623, 20	0.879, 0.000000200, 20	-0.0153, 0.947, 20
Cyano			-0.253, 0.263, 21	0.730, 0.0000641, 21	0.466, 0.0378, 20	0.197, 0.399, 20	-0.112, 0.635, 20
Green				0.345, 0.124, 21	-0.752, 0.000000200, 20	0.536, 0.0150, 20	0.000, 0.997, 20
Chla					0.0331, 0.886, 20	0.432, 0.0565, 20	-0.0779, 0.738, 20
16S						-0.300, 0.194, 20	0.144, 0.538, 20
mcyE							0.254, 0.275, 20

	**T**	**DO**	**NOx**	**NH4**	**TN**	**PO4**	**TP**
Date	-0.802, <0.000001, 20	0.305, 0.187, 20	-0.160, 0.484, 21	-0.0124, 0.953, 21	-0.197, 0.386, 21	-0.164, 0.469, 21	-0.203, 0.370, 21

MC	-0.102, 0.673, 19	0.577, 0.00968, 19	0.0123, 0.957, 19	-0.321, 0.176, 19	0.447, 0.0461, 19	-0.498, 0.0296, 19	-0.108, 0.652, 19
Cyano	0.335, 0.145, 20	0.141, 0.546, 20	0.233, 0.317, 20	-0.194, 0.407, 20	0.560, 0.0104, 20	0.328, 0.155, 20	0.235, 0.314, 20
Green	-0.146, 0.534, 20	0.390, 0.0878, 20	-0.0869, 0.710, 20	-0.223, 0.340, 20	-0.184, 0.433, 20	-0.565, 0.00968, 20	-0.438, 0.0528, 20
Chla	0.282, 0.223, 20	0.256, 0.272, 20	0.322, 0.163, 20	-0.383, 0.0932, 20	0.366, 0.111, 20	-0.167, 0.476, 20	-0.233, 0.317, 20
16S	0.251, 0.294, 19	-0.485, 0.0344, 19	0.279, 0.242, 19	0.0677, 0.776, 19	0.0202, 0.928, 19	0.402, 0.0862, 19	0.150, 0.531, 19
mcyE	-0.116, 0.631, 19	0.554, 0.0137, 19	0.0997, 0.678, 19	-0.208, 0.389, 19	0.402, 0.0862, 19	-0.574, 0.0102, 19	-0.121, 0.615, 19
sxtA	0.0204, 0.928, 19	0.0909, 0.705, 19	-0.0149, 0.951, 19	0.347, 0.142, 19	-0.0872, 0.715, 19	-0.232, 0.334, 19	-0.0538, 0.820, 19
T		-0.320, 0.165, 20	0.0196, 0.932, 20	-0.0302, 0.896, 20	0.390, 0.0878, 20	0.464, 0.0385, 20	0.471, 0.0358, 20
DO			-0.105, 0.653, 20	-0.359, 0.117, 20	0.333, 0.147, 20	-0.326, 0.157, 20	-0.0700, 0.762, 20
NOx				-0.149, 0.513, 21	0.0676, 0.767, 21	-0.404, 0.0683, 21	-0.392, 0.0784, 21
NH4					-0.179, 0.431, 21	0.299, 0.185, 21	0.260, 0.251, 21
TN						0.186, 0.411, 21	0.656, 0.00121, 21
PO4							0.674, 0.000740, 21

	**DIN:DIP**	**TN:TP**
Date	-0.190, 0.402, 21	-0.202, 0.376, 21

MC	0.147, 0.541, 19	0.550, 0.0149, 19
cyano	-0.218, 0.350, 20	0.220, 0.343, 20
Green	0.0634, 0.787, 20	0.305, 0.187, 20
Chla	0.00602, 0.977, 20	0.544, 0.0131, 20
16S	-0.0702, 0.770, 19	-0.248, 0.301, 19
mcyE	0.268, 0.261, 19	0.535, 0.0181, 19
sxtA	0.325, 0.171, 19	-0.111, 0.646, 19
T	-0.187, 0.425, 20	0.0968, 0.681, 20
DO	-0.150, 0.521, 20	0.549, 0.0123, 20
NOx	0.619, 0.00278, 21	0.519, 0.0162, 21
NH4	0.364, 0.103, 21	-0.555, 0.00920, 21
TN	-0.107, 0.637, 21	0.439, 0.0459, 21
	**DIN:DIP**	**TN:TP**
PO4	-0.539, 0.0118, 21	-0.608, 0.00352, 21
TP	-0.342, 0.126, 21	-0.296, 0.189, 21
DIN:DIP		0.273, 0.228,

21
